# Associations between 25 hydroxyvitamin D concentration and spontaneous abortion

**DOI:** 10.1186/s12889-024-19078-5

**Published:** 2024-07-11

**Authors:** Hongping Zhang, Xingru Ding, Xianqing Hu, Yi-Xin Cai, Haiying Chen, Congcong Sun, Jingjing Chen, Xiaoqing Li, Zhenzhen Zheng, Tingting Liao, Na Zhao, Mingchen Zhong, Rujing Fang, Xiu-Feng Huang, Jianqiong Zheng

**Affiliations:** 1 Department of Obstetrics and Gynecology, Wenzhou People’s Hospital, Wenzhou Maternal and Child Health Care Hospital, The Third Clinical Institute Affiliated to Wenzhou Medical University, The Third Affiliated Hospital of Shanghai University, Wenzhou, China; 2 Zhejiang Provincial Clinical Research Center for Pediatric Disease, The Second School of Medicine, The Second Affiliated Hospital and Yuying Children’s Hospital of Wenzhou Medical University, Wenzhou, Zhejiang China; 3 Department of Scientific Research Center, Wenzhou People’s Hospital, Wenzhou Maternal and Child Health Care Hospital, The Third Clinical Institute Affiliated to Wenzhou Medical University, The Third Affiliated Hospital of Shanghai University, Wenzhou, China

**Keywords:** Vitamin D, Abortion, Mendelian randomization

## Abstract

**Background:**

Spontaneous abortion is a common complication of pregnancy that can lead to adverse physical and psychological outcomes for women. Vitamin D is reported to be associated with reproductive functions, whereas its casual effects on abortion remains unclear.

**Materials and methods:**

In this study, a two-sample Mendelian randomization (MR) analysis was performed to systematically assess the causal relationships between serum 25 hydroxyvitamin D [25(OH)D] concentration and the risk of spontaneous abortion. GWAS summary data of 25(OH)D were used as exposure, and data of spontaneous abortion was considered as outcome. A retrospective study was additionally conducted to verify the MR results.

**Results:**

MR estimates showed that a higher 25(OH)D level was potentially associated with decreased risk of spontaneous abortion (IVW, OR = 0.98, 95%CI = 0.90–1.06; MR Egger, OR = 0.94, 95%CI = 0.84–1.05; Weighted median, OR = 0.93, 95%CI = 0.82–1.06; Weighted mode, OR = 0.93, 95%CI = 0.84–1.03), though the P-value was not statistically significant. The retrospective study also produced consistent result of Vitamin D’s protective role to spontaneous abortion. The P-value was very close to statistical significance (*P* = 0.053).

**Conclusions:**

This study reports the potential protective role of serum 25(OH)D concentration to spontaneous abortion, suggesting that increased vitamin D levels may decrease the risk of abortion. Further larger prospective studies and/or even randomized controlled trials are needed to confirm causal relationship between vitamin D and abortion.

**Supplementary Information:**

The online version contains supplementary material available at 10.1186/s12889-024-19078-5.

## Introduction

According to the Royal College of Obstetricians and Gynecologists (RCOG, 2006), spontaneous abortion occurs when a pregnancy ends before 24 weeks of gestation. A global study found that 10–15% of clinical pregnancies result in spontaneous abortion [1]. Spontaneous abortions have a severe impact on a woman’s mental and physical health [2]. Several factors can contribute to these abortions, including maternal immune incompetence, nutritional deficiencies, chromosomal anomalies, infections, and hormonal imbalances [3]. Thus, it is crucial to identify potential protective factors for spontaneous abortion.

Vitamin D is reported to be associated with reproductive functions [4]. Acting as an innate immune modulator and tolerogenic immunological status promoter, vitamin D may help prevent abortion by improving maternal immune tolerance and enhancing implantation [5]. Studies have been reported that active 1,25-dihydroxyvitamin D3 (1,25[OH]_2_D_3_) coupled with its inactive precursor 25OHD_3_, can regulate the functions of decidual immune cells. Moreover, the expression of mRNA encoding an antimicrobial peptide-CAMP increased after the supplementation of 1,25(OH)_2_D_3_ or 25OHD_3_ [6]. In addition, during the implantation window there is an increase in the expression of VDR and HOXA10 protein and subsequently higher serum vitamin D levels, therefore contributing to the establishment of pregnancy and confirming vitamin D’ s status as immune-suppressive factors [7]. Additionally, it is reported that vitamin D could improve the mitochondrial health [8], which if damaged could decrease the reproductive capacity in advanced-age women [9].One study has reported that preconception 25-hydroxyvitamin D [25(OH)D] abundance is associated with higher pregnancy incidence and live birth as well as lower pregnancy loss [10]. Together, these facts suggest that vitamin D might play a significant role in improving pregnancy environment and preventing abortion. Nonetheless, the specific causal relationship is not yet fully understood and thus, further investigations are necessary to elucidate this relationship.

Mendelian randomization (MR) is a research method that uses genetic variants as instrumental variables to explore causal relationships between risk factors and disease outcomes. Recently, for the sake of abundant genetic variants, it has been combined with genome-wide association study (GWAS) summary data that everyone has easy access to [11]. In recent years, MR analysis has become increasingly used in the research of diseases treatment or prevention. Additionally, we found that no MR study revealing vitamin D’ s causal effects on abortion has been carried out, though there have been some studies concerning homocysteine, vitamin B-12, renal functions, diabetes [12], smoking initiation, alcohol drinking and coffee consumption [13], and pregnancy related hypertensive disorders like gestational hypertension and pre-eclampsia [14]. Taken together, we speculate that vitamin D may have positive influences on the prevention of abortion. Thus, this study aims to assess the causal relationship between vitamin D and spontaneous abortion using MR approach. In addition, we also performed a retrospective study to verify their association.

## Methods

### Study design

**Fig. 1:**
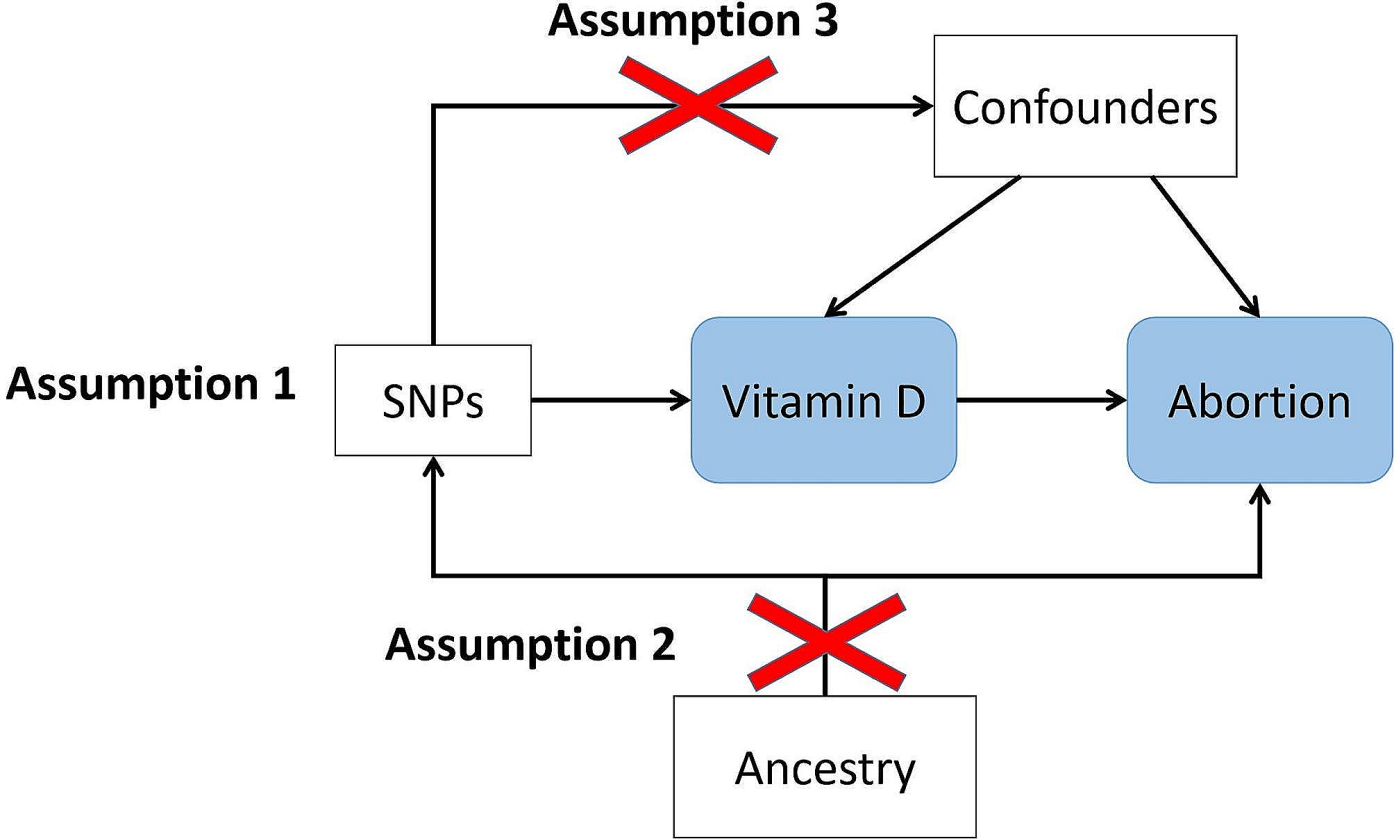
Principles of MR and the assumptions

Our study is a 2-sample MR design, which gains advantages over one-sample MR design by performing measurements between exposure and outcome in separate samples and being able to collaborate with publicly accessible GWAS data [15]. There are three assumptions of 2-sample MR design (Fig. 1). Firstly, genetic variants serving as instruments must have strong relationships with vitamin D. Secondly, those variants are strictly not allowed to correlate with confounders of the exposure-outcome association. Lastly, genetic variants should influence abortion solely by the way of vitamin D.

### GWAS summary statistics

The 25(OH)D-related datasets were obtained from the MRC Integrative Epidemiology Unit (IEU) OpenGWAS database (https://gwas.mrcieu.ac.uk/). To satisfy the first standpoint we have mentioned, the datasets are required to include genetic instruments with statistical significance [16]. Taken together, one dataset met those standards and were adopted for our analyses. The data was generated based on the UK Biobank (UKB) sample, which included over 502,000 individuals. Of these, 449,978 people had their vitamin D 25(OH)D levels measured, and total 25(OH)D (i.e., 25OHD_3_ and 25OHD_2_) was determined by chemiluminescent immunoassay (CLIA) on a Diasorin Liaison®. Those outside of the validated range for the assay (10–375 nmol L^− 1^) were excluded [17]. The spontaneous abortion-related dataset was obtained from FinnGen GWAS database (https://www.finngen.fi/en/access_results) and contained 16,906 cases and 149,622 controls. The summary data of exposure and outcome was ethnically consistent. Additionally, we used the spontaneous abortion-related dataset from the GWAS by Laisk et al. for further validation [18]. The relevant information is shown in Table 1.

**Table 1:** Description of the GWAS summary statistics of traits

Trait	Sample size	Number of cases	Number of controls	Ancestry	Resource
25(OH)D	417,580	NA	NA	European	https://gwas.mrcieu.ac.uk/datasets/ebi-a-GCST90000615/
Spontaneous abortion (from FinnGen)	166,528	16,906	149,622	European	https://www.finngen.fi/en/access_results
Spontaneous abortion (from Laisk et al.-mix)	428,523	69,054	359,469	Multiple races	http://www.geenivaramu.ee/tools/misc_sumstats.zip
Spontaneous abortion (from Laisk et al.-European)	224,105	49,996	174,109	European	http://www.geenivaramu.ee/tools/misc_sumstats.zip

25(OH)D, serum 25 hydroxyvitamin D; NA, not applicable

### MR analysis

Analyses were carried out with the assistance of R package TwoSampleMR (https://github.com/MRCIEU/TwoSampleMR) in the R computing environment [19]. For the sake of the robustness of the connection between SNPs and exposure, the former instrument was adapted only when it was in possession of genome-wide significant variants (*P* < 5 × 10^− 8^). Besides, removal was conducted for variants that correlated with the most significant SNPs (clumping r2 cut-off=0.001, clumping window=10,000 kb), after which we harmonized all included SNPs to ensure the effect allele of a SNP on the exposure equaled to that on the outcome. Then, proxy SNPs with LD of r2 > 0.8 were identified as well as palindromic SNPs were excluded when harmonizing exposure and outcome. Inverse variance–weighted (IVW), a meta-analysis used for estimations of SNP-outcome effects on SNP-exposure effects where intercept is refrained to zero [20], was regarded as the pivotal method with strong statistic power in this study.

### Sensitivity analysis

During the analysis, three more methods were carried out for strengthening the robustness of powerful linkages and potential deviations of the MR assumptions in addition to IVW: (1) MR-Egger regression, a tool which can test for bias from pleiotropy and provide an estimate of the causal effect [21]; (2)Weighted median method, a method that acts as a sensitivity method for Mendelian randomization analyses with multiple genetic variants along with MR-Egger regression [22]; (3)Weighted mode method, a sensitivity analysis used to detect and adjust for pleiotropy as well [23]. Moreover, additional sensitivity analyses were performed, which included the MR pleiotropy residual sum and outlier test, heterogeneity test, and leave-one-out analysis [24]. Furthermore, Steiger filtering was employed to explore the potential impact of reverse associations.

### Retrospective study

This retrospective study was approved by our institutional review board, and informed consent was obtained from all 60 pregnant women enrolled. These pregnant women were recruited from January 2020 to December 2021 at the People’s Hospital of Wenzhou when they registered for antenatal care and delivery. They were divided into two groups: the abortion group (20 participants) included those who had a gestational age of delivery of < 24 weeks, and the term delivery group (40 participants) included those who had a gestational age of delivery between 37 and 42 weeks. At the time of hospitalization for delivery or spontaneous abortion, peripheral whole-blood samples were collected from each group, and the level of 25(OH)D was measured by CLIA. The serum 25(OH)D kit was purchased from Abbott (iSR 61,723, USA). All values are expressed as the median and IQR. Comparison of quantitative variables between the two groups was performed using Wilcoxon test. In this study, differences with *p* < 0.05 were deemed to be statistically significant.

## Results

Effect of vitamin D on spontaneous abortion.

**Fig. 2:**
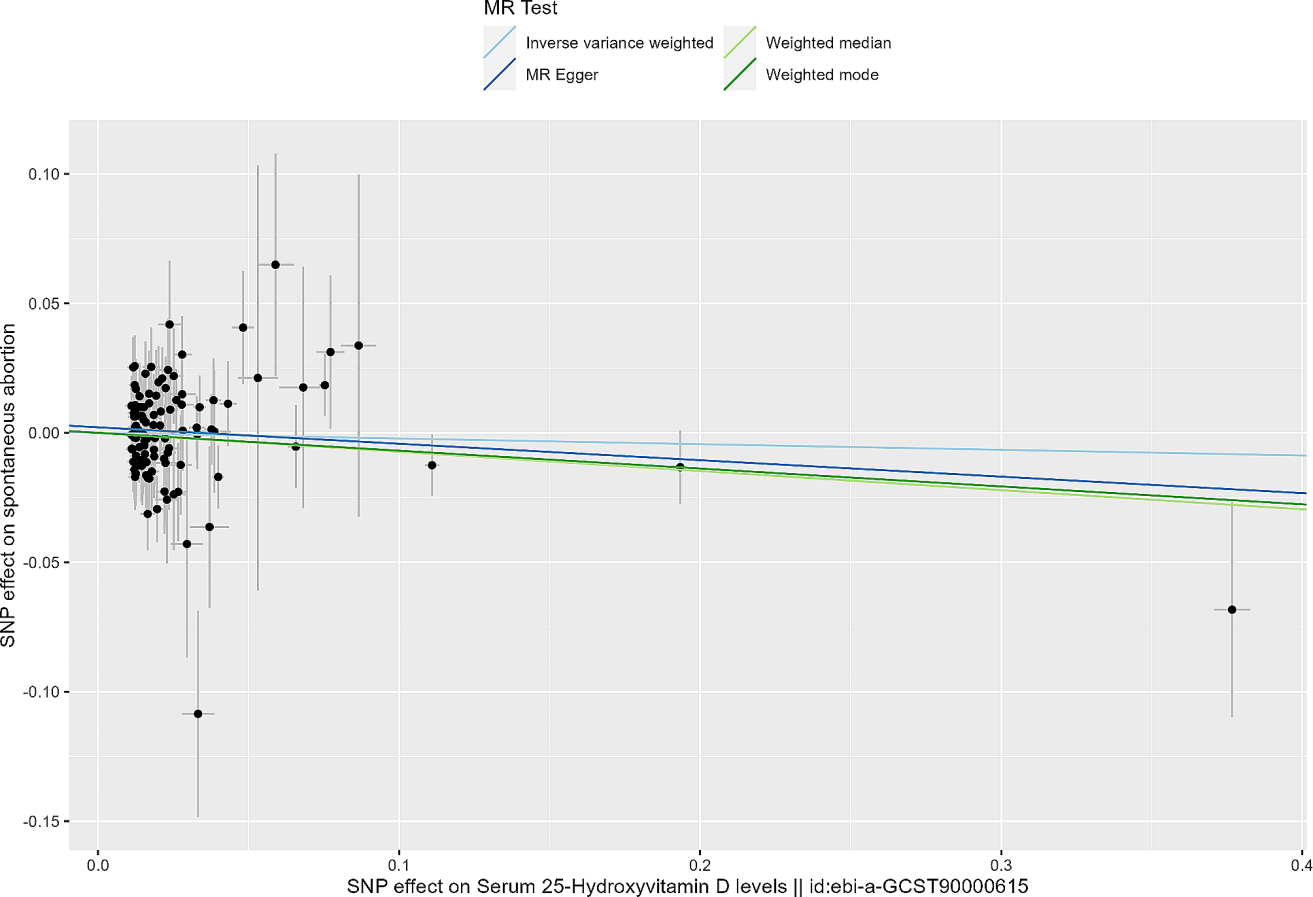
Scatter plots for MR analyses of the effect of vitamin D on spontaneous abortion. The slope of each line corresponding to the estimated MR effect per method

Utilizing 25(OH)D as the exposure and the spontaneous abortion dataset from FinnGen as the outcome, a total of 108 valid genetic instruments were included in the MR analysis (Table S1). According to our analyses, serum 25(OH)D is thought to have potential protective effects on spontaneous abortion with the assistance of such different methods as IVW (odds ratio [OR] = 0.98; 95% confidence interval [CI], 0.90–1.06) and MR Egger (OR = 0.94; 95% CI, 0.84–1.05), though the P-value was > 0.05 (Fig. 2). Weighted median and weighted mode methods also showed the consistent effect direction (Weighted median, OR = 0.93; Weighted mode, OR = 0.93), but P-values were not statistically significant as well (Table 2). The relationship mentioned above has been presented in Fig. 2. Sensitivity analysis confirmed the directionality of the results (Tables S4, S5, S6 and S9).

**Table 2:** Causal effect of 25(OH)D on spontaneous abortion

Exposure/outcome	SNPs	Method	OR	95% CI	*P*-value
25(OH)D /	108	Inverse variance weighted	0.98	0.90–1.06	0.60
Spontaneous abortion (from FinnGen)		MR Egger	0.94	0.84–1.05	0.25
		Weighted median	0.93	0.82–1.06	0.26
		Weighted mode	0.93	0.84–1.03	0.19
25(OH)D /	91	Inverse variance weighted	0.97	0.90–1.05	0.52
Spontaneous abortion (from Laisk et al.-mix)		MR Egger	0.98	0.87–1.10	0.70
		Weighted median	0.97	0.88–1.07	0.55
		Weighted mode	1.04	0.85–1.26	0.71
25(OH)D /	95	Inverse variance weighted	0.98	0.93–1.03	0.36
Spontaneous abortion (from Laisk et al.-European)		MR Egger	0.98	0.91–1.05	0.50
		Weighted median	0.99	0.92–1.06	0.74
		Weighted mode	0.97	0.92–1.03	0.39

25(OH)D, serum 25 hydroxyvitamin D; OR, odds ratio; 95% CI, 95% confidence interval

We then performed validation analysis using a spontaneous abortion-related dataset obtained from the GWAS by Laisk et al. Based on the mixed population samples, a total of 91 valid genetic instruments were included in the MR analysis (Table S3). The MR analysis revealed a potential protective effect of serum 25(OH)D on spontaneous abortion using the IVW method (OR = 0.97; 95% CI, 0.90–1.05). Notably, in the datasets specifically focusing on spontaneous abortion within the European population, a total of 95 valid genetic instruments were included in the MR analysis (Table S2). IVW (OR = 0.98; 95% CI, 0.93–1.03), MR Egger (OR = 0.98; 95% CI, 0.91–1.05), Weighted median (OR = 0.99; 95% CI, 0.92–1.06), and Weighted mode (OR = 0.97; 95% CI, 0.92–1.03) all indicated a protective effect of serum 25(OH)D levels against spontaneous abortion, despite the p-value exceeding 0.05. These results indicated that increased 25(OH)D levels might reduce the risk of spontaneous abortion. The sensitivity analysis also confirmed the reliability of the results (Tables S4, S5, S7, S8 and S9).

### Retrospective study

**Fig. 3:**
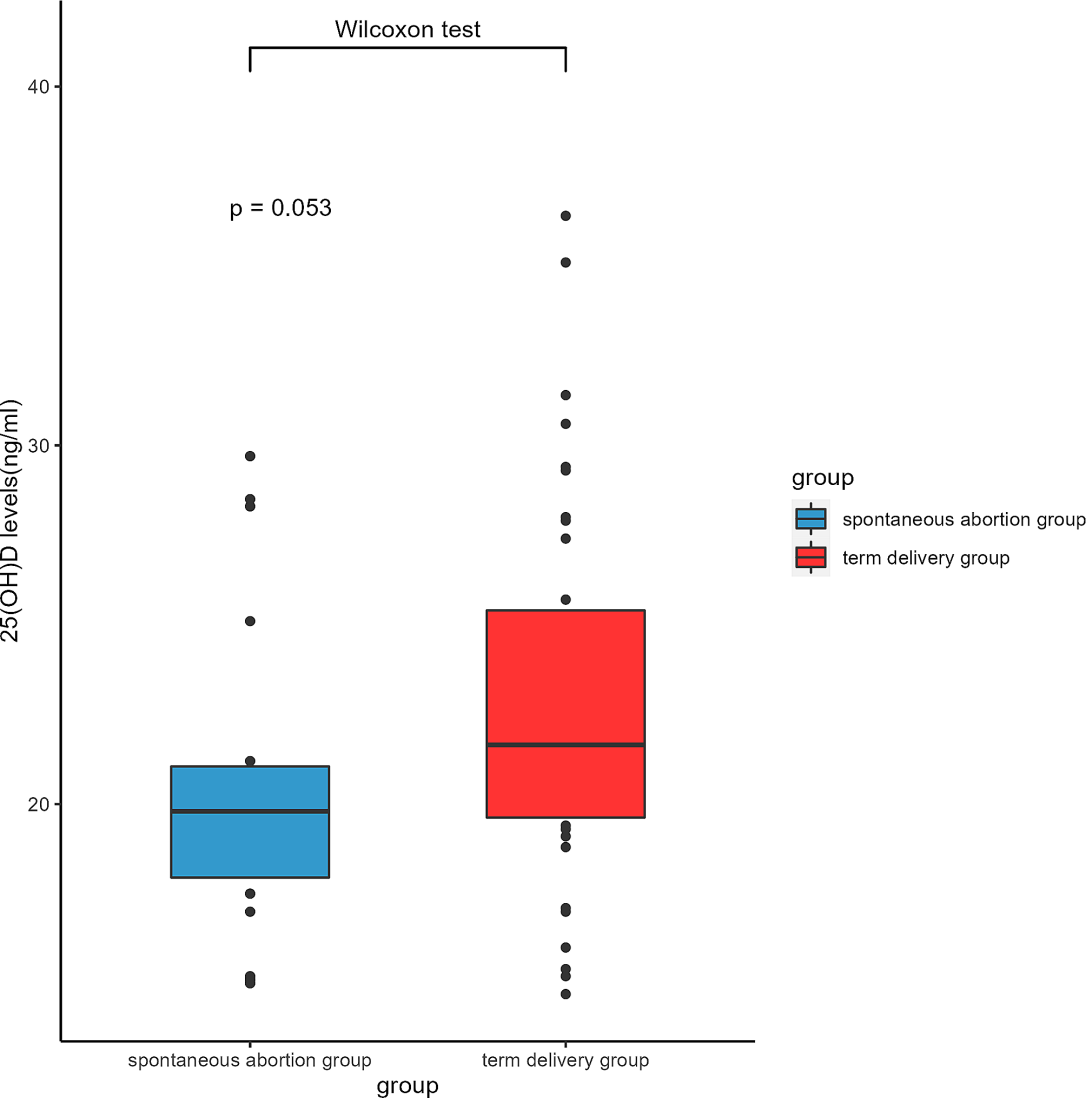
25(OH)D levels in abortion group and term delivery group

To validify the association observed in MR analyses, a retrospective study has been conducted. Our results show that the maternal serum 25(OH)D concentration in term delivery group were higher than those in spontaneous abortion group [21.65(19.63,25.40) VS 19.80(17.95,21.05) ng/mL] (Table 3). However, the difference was not statistically significant (*P* = 0.053, Fig. 3). No statistical differences were observed between the spontaneous abortion group and the term delivery group with respect to maternal age (29.25 ± 3.01 vs. 28.50 ± 2.77), gravidity [2(1,3) vs. 2(1,3)], and parity [1(0,1) vs. 1(0,1)], *P* > 0.05 (Table 3).

**Table 3:** Maternal characteristics of the retrospective study subjects

Variables	spontaneous abortion group	term delivery group	*P*-value
N	20	40	NA
Ages(y)	29.25 ± 3.01	28.50 ± 2.77	0.687
Gravidity	2(1,3)	2(1,3)	0.679
Parity	1(0,1)	1(0,1)	0.844
Specimen collection gestation (W)	21(19.00,22.00)	38(37.00,38.50)	0.00
25(OH)D levels (ng/mL)	19.80(17.95,21.05)	21.65(19.63,25.40)	0.053

NA, not applicable; 25(OH)D, serum 25 hydroxyvitamin D. Normally distributed data are presented as mean ± SD. Independent samples t-test was used to compare quantitative variables between the two groups; Non-normal distribution are presented as Median and IQR. Comparison of quantitative variables between the two groups was performed using Wilcoxon test

## Discussion

The use of MR studies to explore casual relationships is bolstered by the ability to exclude confounders through the random allocation of genetic variants. While this method has been applied to explore casual relationships between other risk factors and abortion, such as homocysteine and smoking [12, 13], our study is the first to analyze the casual effects of vitamin D on abortion using MR analysis. Our results indicate that 25(OH)D, the primary form of vitamin D found in humans, may exert a protective effect against spontaneous abortion. The retrospective study findings, which revealed that serum 25(OH)D concentrations were higher in the term delivery group, were consistent with those produced by MR, further supporting a potential causal relationship between vitamin D and abortion.

The results revealed by this study are consistent with previous discoveries on vitamin D and abortion. For instance, preconception 25-hydroxyvitamin D abundance is associated with higher pregnancy incidence and live birth as well as lower pregnancy loss [10]. Vitamin D deficiency is related to miscarriage [25]. Vitamin D deficiency is thought to accelerate recurrent pregnancy loss but vitamin D pre-pregnancy supplementation can serve as a prevention for unexplained recurrent pregnancy loss [26]. However, the specific causal relationship is not clear and those studies can be inevitably interfered by many confounders including age, diets, education background of pregnant women and so on. Under the circumstances, our MR study, theoretically with minimal reverse causation biases and no heterogeneity and horizontal pleiotropy existing, enjoys an advantage over them. Our findings provide further evidence of a causal relationship between vitamin D and abortion risk.

Here come several clinical implications for abortion risk management and prevention. Pregnant women with lower vitamin concentration levels should be given more priority and caution because vitamin D insufficiency can act as a catalyst for abortion at least based on our study. However, agreed standards of vitamin D investigation, supplementation and management for pregnant women are absent [25]. Moreover, post-abortion care is overall unsatisfactory particularly in such countries as Bangladesh, Haiti, Kenya, Malawi, Namibia, Nepal, Rwanda, Senegal, Tanzania, and Uganda [27]. Yet, post-abortion care is more expensive and demanding than vitamin D management. Taken together, it is of great urgency and necessity for clinicians to come up with a shared standard of vitamin D supplementation for pregnant women, which is believed to enhance the abortion scenarios to a great extent.

There are several limitations in this study. First, level of 25(OH)D was not measured by LC/MS/MS approach. Second, the subjects included in the retrospective study are of Asian ancestry while those used for MR analysis are from European. Third, early prenatal MR studies tend to use the different expressions when describing or conceptualizing their analysis as an application of instrumental variables [28], which could make it more complex to identify the characteristics of each dataset. In addition, due to variations in laboratories and measurement methods employed across different GWAS datasets, only 91 and 95 SNPs from the replication dataset (Laisk et al.) were extracted as IVs. This highlights the necessity for future vitamin D research to leverage larger sample sizes and datasets with more comprehensive information to enhance the robustness of investigations in this domain. Moreover, both the 25(OH)D-related and abortion-related datasets contain data from the UK Biobank, and there is a potential for partial sample overlap, which could potentially introduce bias into the results [29]. Therefore, future research is strongly encouraged, particularly if access to a larger sample size dataset is granted, and ensuring that the exposure and outcome datasets are mutually independent.

## Conclusions

This study reports the potential protective role of serum 25 hydroxyvitamin D concentration to abortion, that is, higher Vitamin D levels can decrease risk of abortion. Further larger prospective studies and/or even randomized controlled trials are needed to confirm causal relationship between vitamin D and abortion.

**Figure legends.**

### Electronic supplementary material

Below is the link to the electronic supplementary material.

Supplementary Material 1

## Data Availability

The GWAS summary statistics that support the findings of this study are included in this published article and its additional material, and the other data are available from the corresponding author on reasonable request.
